# The Past, Present, and Future in Management of Small Renal Masses

**DOI:** 10.1155/2015/364807

**Published:** 2015-09-30

**Authors:** Sarah C. Ha, Haley A. Zlomke, Nicholas Cost, Shandra Wilson

**Affiliations:** University of Colorado School of Medicine, 13001 E. 17th Place, Aurora, CO 80045, USA

## Abstract

Management of small renal masses (SRMs) is currently evolving due to the increased incidence given the ubiquity of cross-sectional imaging. Diagnosing a mass in the early stages theoretically allows for high rates of cure but simultaneously risks overtreatment. New consensus guidelines and treatment modalities are changing frequently. The multitude of information currently available shall be summarized in this review. This summary will detail the historic surgical treatment of renal cell carcinoma with current innovations, the feasibility and utility of biopsy, the efficacy of ablative techniques, active surveillance, and use of biomarkers. We evaluate how technology may be used in approaching the small renal mass in order to decrease morbidity, while keeping rates of overtreatment to a minimum.

## 1. Introduction

The days of “hematuria, an abdominal mass, and weight loss” suggesting the diagnosis of renal cell carcinoma (RCC) are a distant memory. The most common presenting symptom of RCC is no symptom at all. More than half of cases of RCC are now detected incidentally [1]. Incidental findings of renal masses less than 4 cm have increased with the ubiquity of abdominal imaging including the routine use of ultrasound, computerized tomography (CT), and magnetic resonance imaging (MRI) in the work of nonspecific abdominal symptoms [1, 2]. According to the American Urological Association, 85% of patients with RCC that presented asymptomatically were diagnosed with tumors, less than 4 cm in size (Stage I), dictated as small renal masses (SRMs) [1]. Due to an increased incidence and an earlier detection of these masses, the treatment recommendations are evolving. Interestingly, although abdominal imaging has allowed for the downward stage migration of RCC, there has been no impact in the mortality rate [3, 4]. For instance, a SEER review illustrated that mortality rates from kidney cancers have increased 30.9% between 1950 and 2012 with an annual percentage change of 0.4%, even though incidence rates have increased from 7.08 to 15.91 (per 100,000 people) from 1975 to 2012, where majority of tumors represented localized disease [5, 6]. Some suggest that the increased incidence of RCC and other kidney tumors may be attributable to the rise in hypertension and obesity in the US population [5, 7]. In a recent meta-analysis using prospective observational data, the estimated risk of developing RCC increased 24% for men and 34% for women, for every 5 kg/m^2^ increase in body mass index (BMI) [8]. Another investigation discovered that patients with systolic blood pressure (SBP) greater than 160 had a risk of dying from RCC that is nearly double patients with SBP under 120 [5].

With the incidence and mortality trends for RCC continuing in this trajectory, many investigators are searching for novel therapeutic combinations to increase the survival rate of patients with SRMs while keeping the risks of overtreatment and morbidity to a minimum [9]. Reports estimate that, on final pathologic review after excision, 20–40% of the SRMs are benign and thus overtreatment is a legitimate concern [9, 10]. This review aims to highlight some of the benefits and limitations of current treatments and focus on the future for a more definitive diagnosis and treatment of small renal masses (SRMs).

## 2. Small Renal Mass: History and Presentation

SRMs are diverse: from benign to malignant histology, from focal well-defined lesions to those with high anatomic complexity, and from asymptomatic to symptomatic patients [11]. Historically, the patients with RCC presented with a classic triad consisting of hematuria, an abdominal mass, and weight loss, demonstrating later stages of cancerous disease and having life expectancies varying between 10 and 15 months [1, 12, 13]. However, with advanced imaging technology, we are now able to identify these SRMs well before symptoms are present [1]. In one study, SRMs detected with computerized tomography (CT) demonstrated that of the 3,001 patients without symptoms under screening 433 (14.4%) of the patients had at least one cystic or solid renal masses ≥1 cm in diameter [12]. Additionally, other studies report incidental detection of SRMs ranging between 55 and 61% of patients with RCC [1, 14]. Historically, SRMs are classified as masses 4 cm or less, but some studies, particularly those looking at active surveillance (AS), have included masses up to 7 cm in diameter [15–17]. When we use the term “small renal mass” or “SRM,” in this review, we are specifically referring to masses equal to or less than 4 cm.

RCC is the ninth most common cancer worldwide [12]. A study conducted by Frank et al. made it evident that of 947 SRMs tumor samples of less than 4 cm in diameter 726 (76.7%) were classified as RCC [18]. The predictability of growth, complexity, and prognosis can be complicated and incorporating the patient's frailty and comorbidities is becoming increasingly important in creating a feasible treatment plan [12]. RCC survival correlated with TNM staging, where stage I RCC patients have a five-year disease-specific survival rate between 80 and 95%, and patients with stage IV disease have a five-year disease-specific survival rate of less than 10%, with a median of 10–15 months [12, 19]. However, with immunomodulatory treatment and targeted therapies, the median overall survival of stage IV RCC is now beyond two years [19]. With the advent of eight new FDA-approved agents for RCC therapy in the last few years, the 5-year survival rate for RCC has increased dramatically, from 52.1% in 1975 to 73.2% between 2005 and 2011 [6].

## 3. Surgical Excision

Radical nephrectomy has long been the mainstay treatment for RCC for over 50 years [20, 21]. Within the past decade, there has been a shift of surgical technique with an increasing utilization for elective nephron-sparing surgery. For the first time in 2009, the percentage of partial nephrectomies exceeded the percentage of radical nephrectomies performed on patients with renal masses less than 4 cm [20]. In one well-quoted study, 5994 patients who had been treated with partial nephrectomy were compared to patients who had undergone radical nephrectomy [20]. The 3-year and 5-year overall survival probabilities were 83% and 72% in patients who had been treated with radical nephrectomy and 90% and 81%, respectively, in those who received nephron-sparing surgery [20]. Although there is always a concern about selection bias, certainly this gives patients and surgeons confidence in this choice as patients have more and more comorbidities that can threaten their renal health. It has been shown that patients undergoing nephron-sparing surgery (NSS) experience the expected improved renal function but also a lower risk of cardiovascular events associated with chronic kidney disease [4, 22]. Additionally, there is growing concern for increased proteinuria in patients, after nephrectomy, as a side effect of glomerular hyperfiltration due to adaptation or compensation after nephrectomy [23]. This is assumed to be less with NSS [23]. A study by Choi et al. discussed the risk of progression or the development of chronic kidney disease postoperative nephrectomy, where 798 of the 1502 patients who had radical nephrectomies (53.1%) went on to develop renal failure, whereas only 133 of the 952 patients who had partial nephrectomies (13.9%) developed renal failure [23]. This study was also able to show that hyperfiltration is more common after radical nephrectomy, with patients undergoing radical nephrectomies having higher estimated glomerular filtration rates (EGFR) postoperatively, which leads to proteinuria and other complications of renal failure [23]. As such, the current recommendation by the American Urological Association and the European Association of Urology to excise small renal masses less than 4 cm is through partial nephrectomy [24, 25].

On the other hand, partial nephrectomy is more challenging technically as compared to radical nephrectomy. One study, using the Clavien-Dindo postoperative complication classification system, demonstrated that minor and major complications for partial nephrectomies are as high as 26.7% and 11.5%, respectively [26]. This study also found that major complications correlated with the heightened renal tumor anatomical complexity [26], suggesting that the complexity of excision and complications associated with it are attributable to the anatomical characteristics of the renal tumor [4]. Furthermore, a study by the EORTC randomized patients with small renal masses (they were defined as ≤5 cm) for partial or radical nephrectomy of 541 patients from 45 different institutions illustrated the impact of nephrectomies on eGFR subsequently to the surgery [27]. With a median follow-up of 6.7 years for EGFR, patients that underwent NSS compared to radical nephrectomy had a substantial reduction of incidences of renal dysfunction, yet the beneficial impact of EGFR for the NSS group of patients did not result in improved overall survival over a mean follow-up of 9.3 years for all-cause mortality [27]. These studies may be helpful in selecting appropriate patients for diverse types of surgery or laparoscopic/robotic surgery in particular. Partial nephrectomy has created interest due to studies demonstrating increase in renal function and decrease in overall health complications related to partial resection; but with current studies and potential selection bias, there needs to be further research into individualized surgical procedures or guidelines for specific populations to improve overall survival and quality of life and providers need to be cautious in the selection of the best-fit procedure for their patients based on their comorbidities and lifestyles.

## 4. Biopsy

A renal mass biopsy (RMB) has historically been performed for proof of malignancy and documentation of tumor grade and to clarify histology [28, 29]. Since it has been shown that 20–40% of SRMs are in fact benign, it is appropriate to confirm the diagnosis in patients with renal masses under 4 cm [10]. A RMB may also be performed in those undergoing AS and chemotherapy and candidates for ablative therapies. Despite its limited utilization (only 20.7% of SRMs underwent biopsy from 1992 to 2007) [30, 31], biopsy for RMB has been demonstrated as beneficial in diagnosing tumors and avoiding the overtreatment of benign masses [32]. It has also been shown that a RMB is relatively safe in terms of tumor reseeding because studies show that reseeding of the tumor is extremely rare, occurring between 0.0 and 0.009% of the time [29, 33, 34]. Richard et al. showed that a RMB has a 93% agreement with final surgical pathology [32]. In the same study, about half (45%) of the nondiagnostic specimens (which was only 10% of the total) were rebiopsied and 83% came back as diagnostic. Multiple cores likely increase the accuracy of the biopsy beyond the 93%. New studies suggest that clear cell renal cell carcinomas (ccRCC) have intratumor heterogeneity and RMB may underestimate the grade that the mass truly presents [11, 28, 35]. A recent study showed that 93% of high-grade specimens (Fuhrman score 3-4) also had low-grade components (Fuhrman score 1-2) [11]. A multiple core biopsy may be considered to capture the heterogeneous tissue characteristics. More interestingly, multicore SRM biopsies should perhaps be performed for future molecular or genetic targeted molecular therapy [28].

Although the incidences of SRMs have been increasing 2 to 2.5% each year, the mortality rate for kidney cancers has remained relatively stable [13]. The rates of surgical interventions have also paralleled the incidence of detection [4, 13]. Wendler et al. report that the estimated number of surgically removed benign renal masses increased by 82% from 3,098 in 2000 to 5,624 in 2009 [10]. The incidence of confirmed benign renal masses is higher in the United States compared to other countries and is thought to be related to the overutilization of imaging [10]. Therefore, the concept of RMB is especially important in order to prevent unnecessary surgical procedures and their inherent complications.

## 5. Ablation

Ablative therapies that spare nephrons and potentially avoid general anesthesia are gaining popularity. These therapies have generally been recommended for those with multiple comorbidities or those that cannot undergo surgery. However, some series with short- and intermediate-term follow-up find that these therapies rival partial nephrectomy in terms of oncologic outcome in the treatment for SRMs [36]. Most ablative therapies are performed laparoscopically or with CT guidance.

In a recent study by Larcher et al. they investigated renal function outcomes of 2,850 individuals who underwent local tumor ablation (specific modality was not described) versus a partial nephrectomy [37]. They investigated short-term outcomes based on acute kidney injury and any dialysis 30 days after treatment. They also reported long-term outcomes 5 years later based on chronic kidney disease defined by GFR, end-stage renal disease, kidney failure necessitating replacement, hemodialysis rates, and anemia. They found no significant difference between the two treatments in any parameter in the long-term, 5-year follow-up. However, local tumor ablation did have a benefit in the short term 30 days after treatment regarding acute kidney injury when compared to partial nephrectomy; ablation had a 4.6% incidence of acute kidney injury compared to 9.4% of those who underwent a partial nephrectomy [37].

The most studied ablative treatments include radiofrequency ablation (RFA) and cryoablation [38]. New therapies include microwave ablation, irreversible electroporation, and high-intensity focused ultrasound [39]. Studies have been done to compare RFA and cryoablation. In 2012, a meta-analysis reviewed thirty-one case series to determine the clinical efficacy and complication rates between these two most common ablative therapies. Most cases were highly selected with a clinical efficacy of 89% for cryoablation with an 18.1 average month follow-up and 90% for RFA with a 17.9 average month follow-up [36]. This report observed no statistically significant difference between the two for either efficacy or complications.

Due to the ambiguity of which modality to choose, a cost analysis was performed that investigated current nonsurgical treatments in one study: AS, RFA, and cryoablation [40]. The analysis compared quality-adjusted life expectancy with the cost of each therapy. Not surprisingly, the results showed that AS with later cryoablation, if needed, was most effective. Percutaneous cryoablation was more cost effective than the percutaneous RFA. Immediate cryoablation had cost of $3,010 more with similar quality-adjusted life expectancies than AS and delayed cryoablation. Meanwhile RFA had costs of $3,231–$6,398 and reduced quality-adjusted life expectancy compared to AS plus delayed cryoablation. With this data, there is a suggestion of slight preference for cryoablation given health care economics, but, independent of cost, the deciding factor seems to center around operator preference.

A current limitation to ablative therapies is the absence of long-term follow-up data. RFA, for example, was only first described in the literature in 1997 [39, 41]. As data continues to emerge regarding the long-term effects of current ablative therapies, novel therapies continue to develop. Microwave ablation, historically used for liver masses, is under investigation for RCC treatment. It has been shown to have similar outcomes compared to partial nephrectomy with regard to surgical, oncological, and functional outcomes with a 36-month follow-up [39, 42]. Irreversible electroporation (IRE) for renal masses is another ablative therapy under investigation utilizing the electric pulses between two electrodes as the mechanism. Currently, a prospective study is evaluating the efficacy and safety of this treatment after four weeks [43]. Another potential limitation is the difficulty of the procedure and the requirement of having trained and highly skilled operators. Nonetheless, the ability to treat these masses in real time during real-time scanning in an open CT scanner under sedation is an advance over “2D” laparoscopic ablation under general anesthesia.

## 6. Active Surveillance

With the growing number of (very) small renal masses identified on imaging, the development guidelines for AS have been important. Several studies have reported the overtreatment of SRMs with the current spike in discoveries of benign renal masses, raising concerns regarding the potential for unnecessary surgical costs and complications [44–47]. There has been a growing body of evidence deeming AS in very select patients as a competitive alternative to surgical excision due to the comparable survival outcomes [46, 47]. In an analysis using the DISSRM (Delayed Intervention and Surveillance for Small Renal Masses, a multi-institutional registry) Registry, patients were counseled between primary intervention (nephrectomy or ablation) and AS on the risks and benefits and then given the option to choose [46]. In the trial, 21 patients of the 274 in the AS category underwent delayed intervention, where 15 of the patients chose elective intervention and 6 had imperative indications for intervention [46]. This study was able to show that the cancer free survival at 5 years for primary intervention and AS was 99% and 100%, respectively, demonstrating that AS should be part of the mainstay conversations between urologists and their patients on treatment options [46].

Generally, patients selected in the studies for AS for SRMs are imaged every 6 months [44, 46]. Indications for more aggressive therapy (the development of symptoms or growth of the mass) were only realized by 2% to 6% of patients, depending on the study and cohort size [44, 46]. Overall, patients in the DISSRM study showed that 40% of patients elected for AS once they knew that it was an option, demonstrating that patients could elect for this alternative if they were given more counseling prior to the intervention [46].

In another study, patients with multiple comorbid conditions also often opt for AS (with an isolated renal mass less than 3.5 cm) [47]. In this study of 29 such patients, only 4 underwent delayed surgical intervention with 1 due to growth of renal mass and 3 due to elective delayed intervention [47]. None of the patients developed metastatic disease in the duration of the study and two of the patients died of other causes (1 from metastatic prostate cancer and the other from thyroid cancer) [47]. In general, it appears with this approach that the risk of death from RCC is low [47].

Another study advocates the use of AS for elderly patients, because active RCC treatment has not been shown to decrease cancer mortality rates in this patient population [15]. In a cohort of 110 patients with a median age of 81, 43% of the patients showed a stable disease without progression and 31% of the patients were deceased at the end of the study with none of the patients' deaths contributable to RCC [15, 19].

In terms of the natural history of mass growth, a recent review of 70 patients demonstrated a mean growth rate of 0.17 cm/year [16]. In this population, 31% of the masses did not grow at all during the follow-up period ranging from 12 to 112 months. While 10% of the masses were eventually operated on via NSS because of rapid growth, the delayed treatment did not compromise the overall outcome. This study and others have demonstrated that larger masses at time of diagnosis grow faster than smaller lesions [16, 48]. Mason et al. observed that masses <2.45 cm grew at a rate of 0.13 cm/year while those ≥2.45 cm at diagnosis grew significantly faster at 0.40 cm/year [48]. This suggests that the size of the tumor when it is first discovered could be one of the variables utilized to help stratify reasonable candidates for AS. Performing surveillance with serial imaging may be an especially safe and effective approach in those with the smallest masses particularly in patients with multiple comorbidities or the very elderly [15, 16, 48]. In fact, one study has demonstrated that in the very elderly (average age of 78) with slightly larger renal masses (average 4.25 cm at diagnosis) and a more comorbidity they are more likely to die from causes other than RCC [49].

Although conservative management of suspected RCC is growing in popularity, it should be emphasized that progression is still a possibility and thus close follow-up is indicated, because the tumor may not be necessarily at a 100% secure tumor size and some SRMs may albergate high-grade necrosis or other unfavorable features [28, 35]. While AS represents a reasonable alternative to primary curative intervention such as nephrectomy or ablation, this type of treatment still necessitates careful patient selection, careful management, patient compliance, and ongoing follow-up.

## 7. Biomarkers

A recent study by Morrissey et al., 2015, investigated two urine biomarkers, aquaporin 1 (AQP1) and perilipin 2 (PLIN2) [50]. These markers have been shown to be elevated in clear cell and papillary RCC [50, 51]. This study assessed the sensitivity and specificity of these biomarkers, normalized to urine creatinine. They found a 99% sensitivity and a 100% specificity in distinguishing between RCCs and benign renal masses. Interestingly, the level of biomarker in the urine positively correlated with the size of the clear cell or papillary RCC and decreased significantly after the mass was removed [50]. These levels were not confounded by the size of a tumor that was not clear cell or papillary RCC. One limitation of this potential screen however is the involved process of the western blot procedure and the likely costs associated with it. Otherwise, this test holds great promise for screening patients with SRM to aid in the differential diagnosis.

Additional biomarkers have been utilized, such as CAIX, COX-2, and VEGF for prognostic and target therapy [52]. Carbonic anhydrase IX (CAIX) is expressed in high levels in malignant RCC tumor cells, with little to no expression in normal renal tissues or benign masses [53]. CAIX catalyzes the reaction CO_2_ + H_2_O↔HCO_3_^−^ + H^+^ to help regulate the intracellular and extracellular pH in the proximal renal tubules [53, 54]. As such, CAIX is a prime target for imaging clear cell RCC lesions. In 1986, a monoclonal antibody,* girentuximab*, was discovered to target a specific antigen RCC tumor cells. The antibody has no severe side effects and little to no allergic cross-reactivity [53]. There are different studies in process that evaluate the use of* girentuximab* infused with different radionuclide such as indium-111 or Lutetium-177 labeling to try to differentiate malignant tumors from benign masses [53, 55]. As such, this would represent an RCC specific functional imaging modality.

Patients with high CAIX have better responses to interleukin-2 (IL-2) therapy, and patients with high COX-2 have improved responses to interferon alpha (IFN-*α*) therapy. Similarly, patients with higher VEGF staining show more impressive responses to anti-VEGF agents [52]. Overall, the use of biomarkers has a great potential not only for detection and differentiation but also for the appropriate selection of specific targeted therapies. Most recently, because of the impressive data on the use of checkpoint inhibition and immunomodulation in RCC, there is great interest identifying checkpoint biomarkers that can predict response in patients with metastatic renal cell carcinoma [56]. Overall, CAIX, COX-2, and VEGF biomarkers are primarily utilized for more advanced stages of RCC and there is a significant lack of biomarkers available for SRMs; it would be interesting to see future research performed to see the significance of such biomarkers in SRM or if other biomarkers are more colloquial.

## 8. Conclusion

What does the future hold? As Wayne Gretzky once said, “Skate to where the puck is going, not to where it has been.” So we ask what is the outlook for SRMs in 20 years? With the rapid advancement over the past decade in RCC the possibilities are exciting to imagine. Without question, technological advances will bring about novel ways to diagnose, treat, and monitor these patients. It is the duty of practitioners to stay abreast of these changes to provide the optimal care, maximizing cure and minimizing morbidity, to their patients.

## 9. Materials and Methods

A broad analysis of the literature was considered when the authors were invited to write a systematic review. All articles between 2010 and 2015 were considered and the period of research was performed in a one-month period. The data investigated was executed in PubMed using MeSH terms and all literature considered being in English. A special emphasis was placed on articles published in high impact journals and prospective randomized trials and subjects that were germane to this review.

## Abbreviations

RCC: Renal cell carcinoma
SRM: Small renal mass
NSS: Nephron-sparing surgery
RMB: Renal mass biopsy
ccRCC: Clear cell renal cell carcinoma
AS: Active surveillance
AQP1: Aquaporin 1
PLIN2: Perilipin 2.

## References

[1] Jayson M., Sanders H. (1998). Increased incidence of serendipitously discovered renal cell carcinoma. *Urology*.

[2] Kane C. J., Mallin K., Ritchey J., Cooperberg M. R., Carroll P. R. (2008). Renal cell cancer stage migration: analysis of the national cancer data base. *Cancer*.

[3] Hollingsworth J. M., Miller D. C., Daignault S., Hollenbeck B. K. (2006). Rising incidence of small renal masses: a need to reassess treatment effect. *Journal of the National Cancer Institute*.

[4] Tomaszewski J. J., Smaldone M. C., Uzzo R. G., Kutikov A. (2015). Is radical nephrectomy a legitimate therapeutic option in patients with renal masses amenable to nephron-sparing surgery?. *BJU International*.

[5] Chow W.-H., Devesa S. S. (2008). Contemporary epidemiology of renal cell cancer. *Cancer Journal*.

[6] Howlader N., Noone A., Krapcho M. (2015). *Seer Cancer Statistics Review*.

[7] Sanfilippo K. M., McTigue K. M., Fidler C. J. (2014). Hypertension and obesity and the risk of kidney cancer in 2 large cohorts of US men and women. *Hypertension*.

[8] Renehan A. G., Tyson M., Egger M., Heller R. F., Zwahlen M. (2008). Body-mass index and incidence of cancer: a systematic review and meta-analysis of prospective observational studies. *The Lancet*.

[9] Johnson D. C., Vukina J., Smith A. B. (2015). Preoperatively misclassified, surgically removed benign renal masses: a systematic review of surgical series and united states population level burden estimate. *The Journal of Urology*.

[10] Wendler J. J., Porsch M., Nitschke S. (2015). A prospective Phase 2a pilot study investigating focal percutaneous irreversible electroporation (IRE) ablation by NanoKnife in patients with localised renal cell carcinoma (RCC) with delayed interval tumour resection (IRENE trial). *Contemporary Clinical Trials*.

[11] Ball M. W., Bezerra S. M., Gorin M. A. (2015). Grade heterogeneity in small renal masses: potential implications for renal mass biopsy. *The Journal of Urology*.

[12] Jonasch E., Gao J., Rathmell W. K. (2014). Renal cell carcinoma. *British Medical Journal*.

[13] Rendon R. A., Stanietzky N., Panzarella T. (2000). The natural history of small renal masses. *The Journal of Urology*.

[14] Rabjerg M., Mikkelsen M. N., Walter S., Marcussen N. (2014). Incidental renal neoplasms: is there a need for routine screening? A Danish single-center epidemiological study. *APMIS*.

[15] Abouassaly R., Lane B. R., Novick A. C. (2008). Active surveillance of renal masses in elderly patients. *Journal of Urology*.

[16] Bahouth Z., Halachmi S., Meyer G., Avitan O., Moskovitz B., Nativ O. (2015). The natural history and predictors for intervention in patients with small renal mass undergoing active surveillance. *Advances in Urology*.

[17] Clavien P. A., Barkun J., De Oliveira M. L. (2009). The clavien-dindo classification of surgical complications: Five-year experience. *Annals of Surgery*.

[18] Frank I., Blute M. L., Cheville J. C., Lohse C. M., Weaver A. L., Zincke H. (2003). Solid renal tumors: an analysis of pathological features related to tumor size. *Journal of Urology*.

[19] O'Connor S. D., Pickhardt P. J., Kim D. H., Oliva M. R., Silverman S. G. (2011). Incidental finding of renal masses at unenhanced CT: prevalence and analysis of features for guiding management. *American Journal of Roentgenology*.

[20] Huang W. C., Atoria C. L., Bjurlin M. (2015). Management of small kidney cancers in the new millennium: contemporary trends and outcomes in a population-based cohort. *JAMA Surgery*.

[21] Meeks J. J., Gonzalez C. M. (2015). Standard of care for small renal masses in the 21st century. *JAMA Surgery*.

[22] Sun M., Bianchi M., Hansen J. (2012). Chronic kidney disease after nephrectomy in patients with small renal masses: a retrospective observational analysis. *European Urology*.

[23] Choi Y. S., Park Y. H., Kim Y.-J., Kang S. H., Byun S.-S., Hong S.-H. (2014). Predictive factors for the development of chronic renal insufficiency after renal surgery: a multicenter study. *International Urology and Nephrology*.

[24] Campbell S. C., Novick A. C., Belldegrun A. (2009). Guideline for management of the clinical T1 renal mass. *Journal of Urology*.

[25] Ljungberg B., Bensalah K., Canfield S. (2015). Eau guidelines on renal cell carcinoma: 2014 update. *European Urology*.

[26] Simhan J., Smaldone M. C., Tsai K. J. (2011). Objective measures of renal mass anatomic complexity predict rates of major complications following partial nephrectomy. *European Urology*.

[27] Scosyrev E., Messing E. M., Sylvester R., Campbell S., Van Poppel H. (2014). Renal function after nephron-sparing surgery versus radical nephrectomy: results from EORTC randomized trial 30904. *European Urology*.

[28] Tomaszewski J. J., Uzzo R. G., Smaldone M. C. (2014). Heterogeneity and renal mass biopsy: a review of its role and reliability. *Cancer Biology and Medicine*.

[29] Volpe A., Finelli A., Gill I. S. (2012). Rationale for percutaneous biopsy and histologic characterisation of renal tumours. *European Urology*.

[30] Breau R. H., Crispen P. L., Jenkins S. M., Blute M. L., Leibovich B. C. (2011). Treatment of patients with small renal masses: a survey of the american urological association. *The Journal of Urology*.

[31] Leppert J. T., Hanley J., Wagner T. H. (2014). Utilization of renal mass biopsy in patients with renal cell carcinoma. *Urology*.

[32] Richard P. O., Jewett M. A., Bhatt J. R. (2015). Renal tumor biopsy for small renal masses: a single-center 13-year experience. *European Urology*.

[33] Smith E. H. (1991). Complications of percutaneous abdominal fine-needle biopsy: review. *Radiology*.

[34] Klatte T. (2012). The contemporary role of renal tumor biopsy. *European Urology*.

[35] Conti A., Santoni M., Sotte V. (2015). Small renal masses in the era of personalized medicine: tumor heterogeneity, growth kinetics, and risk of metastasis. *Urologic Oncology: Seminars and Original Investigations*.

[36] El Dib R., Touma N. J., Kapoor A. (2012). Cryoablation vs radiofrequency ablation for the treatment of renal cell carcinoma: a meta-analysis of case series studies. *BJU International*.

[37] Larcher A., Meskawi M., Valdivieso R. (2015). Comparison of renal function detriments after local tumor ablation or partial nephrectomy for renal cell carcinoma. *World Journal of Urology*.

[38] Pieper C. C., Fischer S., Strunk H. (2015). Percutaneous CT-guided radiofrequency ablation of solitary small renal masses: a single center experience. *RöFo*.

[39] Khiatani V., Dixon R. G. (2014). Renal ablation update. *Seminars in Interventional Radiology*.

[40] Bhan S. N., Pautler S. E., Shayegan B., Voss M. D., Goeree R. A., You J. J. (2013). Active surveillance, radiofrequency ablation, or cryoablation for the nonsurgical management of a small renal mass: a cost-utility analysis. *Annals of Surgical Oncology*.

[41] Zlotta A. R., Wildschutz T., Raviv G. (1997). Radiofrequency interstitial tumor ablation (RITA) is a possible new modality for treatment of renal cancer: ex vivo and in vivo experience. *Journal of Endourology*.

[42] Guan W., Bai J., Liu J. (2012). Microwave ablation versus partial nephrectomy for small renal tumors: intermediate-term results. *Journal of Surgical Oncology*.

[43] Wagstaff P. G., de Bruin D. M., Zondervan P. J. (2015). The efficacy and safety of irreversible electroporation for the ablation of renal masses: a prospective, human, in-vivo study protocoll. *BMC Cancer*.

[44] Brunocilla E., Borghesi M., Monti C., Schiavina R., Martorana G. (2013). Surveillance for small renal masses: retrospective analysis of a cohort of 42 patients with long-term follow-up. *International Urology and Nephrology*.

[45] Morrissey J. J., London A. N., Luo J., Kharasch E. D. (2010). Urinary biomarkers for the early diagnosis of kidney cancer. *Mayo Clinic Proceedings*.

[46] Pierorazio P. M., Johnson M. H., Ball M. W. (2015). Five-year analysis of a multi-institutional prospective clinical trial of delayed intervention and surveillance for small renal masses: the DISSRM Registry. *European Urology*.

[47] Wehle M. J., Thiel D. D., Petrou S. P., Young P. R., Frank I., Karsteadt N. (2004). Conservative management of incidental contrast-enhancing renal masses as safe alternative to invasive therapy. *Urology*.

[48] Mason R. J., Abdolell M., Trottier G. (2011). Growth kinetics of renal masses: analysis of a prospective cohort of patients undergoing active surveillance. *European Urology*.

[49] O'Connor K. M., Davis N., Lennon G. M., Quinlan D. M., Mulvin D. W. (2009). Can we avoid surgery in elderly patients with renal masses by using the Charlson comorbidity index?. *BJU International*.

[50] Morrissey J. J., Mobley J., Figenshau R. S., Vetter J., Bhayani S., Kharasch E. D. (2015). Urine aquaporin 1 and perilipin 2 differentiate renal carcinomas from other imaged renal masses and bladder and prostate cancer. *Mayo Clinic Proceedings*.

[51] Tøndel C., Vikse B. E., Bostad L., Svarstad E. (2012). Safety and complications of percutaneous kidney biopsies in 715 children and 8573 adults in Norway 1988–2010. *Clinical Journal of the American Society of Nephrology*.

[52] Kim H. S., Kim W. S., Park S. H. (2010). Molecular biomarkers for advanced renal cell carcinoma: implications for prognosis and therapy. *Urologic Oncology*.

[53] Muselaers C. H. J., Boerman O. C., Oosterwijk E., Langenhuijsen J. F., Oyen W. J. G., Mulders P. F. A. (2013). Indium-111-labeled girentuximab immunospect as a diagnostic tool in clear cell renal cell carcinoma. *European Urology*.

[54] Wykoff C. C., Beasley N. J. P., Watson P. H. (2000). Hypoxia-inducible expression of tumor-associated carbonic anhydrases. *Cancer Research*.

[55] Stillebroer A. B., Boerman O. C., Desar I. M. E. (2013). Phase 1 radioimmunotherapy study with lutetium 177-labeled anti-carbonic anhydrase ix monoclonal antibody girentuximab in patients with advanced renal cell carcinoma. *European Urology*.

[56] Monzon J. G., Heng D. Y. C. (2014). Management of metastatic kidney cancer in the era of personalized medicine. *Critical Reviews in Clinical Laboratory Sciences*.

